# Pacifier Overuse and Conceptual Relations of Abstract and Emotional Concepts

**DOI:** 10.3389/fpsyg.2017.02014

**Published:** 2017-12-01

**Authors:** Laura Barca, Claudia Mazzuca, Anna M. Borghi

**Affiliations:** ^1^Institute of Cognitive Science and Technologies, Italian National Research Council (CNR), Rome, Italy; ^2^Department of Philosophy and Communication, University of Bologna, Bologna, Italy; ^3^Department of Dynamic and Clinical Psychology, Sapienza University of Rome, Rome, Italy

**Keywords:** abstract concepts, pacifier, conceptual relations, facial mimicry, social interaction, embodied cognition

## Abstract

This study explores the impact of the extensive use of an oral device since infancy (pacifier) on the acquisition of concrete, abstract, and emotional concepts. While recent evidence showed a negative relation between pacifier use and children's emotional competence (Niedenthal et al., 2012), the possible interaction between use of pacifier and processing of emotional and abstract language has not been investigated. According to recent theories, while all concepts are grounded in sensorimotor experience, abstract concepts activate linguistic and social information more than concrete ones. Specifically, the Words As Social Tools (WAT) proposal predicts that the simulation of their meaning leads to an activation of the mouth (Borghi and Binkofski, 2014; Borghi and Zarcone, 2016). Since the pacifier affects facial mimicry forcing mouth muscles into a static position, we hypothesize its possible interference on acquisition/consolidation of abstract emotional and abstract not-emotional concepts, which are mainly conveyed during social and linguistic interactions, than of concrete concepts. Fifty-nine first grade children, with a history of different frequency of pacifier use, provided oral definitions of the meaning of abstract not-emotional, abstract emotional, and concrete words. Main effect of concept type emerged, with higher accuracy in defining concrete and abstract emotional concepts with respect to abstract not-emotional concepts, independently from pacifier use. Accuracy in definitions was not influenced by the use of pacifier, but correspondence and hierarchical clustering analyses suggest that the use of pacifier differently modulates the conceptual relations elicited by abstract emotional and abstract not-emotional. While the majority of the children produced a similar pattern of conceptual relations, analyses on the few (6) children who overused the pacifier (for more than 3 years) showed that they tend to distinguish less clearly between concrete and abstract emotional concepts and between concrete and abstract not-emotional concepts than children who did not use it (5) or used it for short (17). As to the conceptual relations they produced, children who overused the pacifier tended to refer less to their experience and to social and emotional situations, use more exemplifications and functional relations, and less free associations.

## Introduction

### Embodied and grounded views and abstract concepts representation

The difficulty in acquiring and processing abstract concepts, such as “freedom” and “phantasy” is widely recognized: they have been named “hard words” (Gleitman et al., 2005; Gentner, 2006)! The way in which we represent abstract concepts has become hotly debated in the last years, also due to the growing interest for them in the context of embodied and grounded (EG) views of cognition (for overviews, see Dove, 2011, 2016; Pecher et al., 2011; Tomasino and Rumiati, 2013; Borghi and Binkofski, 2014; Reilly et al., 2016; for overviews showing the importance of abstract concepts for EG views, see Kiefer and Pulvermüller, 2012; Borghi et al., 2017). According to EG views, language comprehension consists in simulating word meaning re-enacting previous experiences with words' referents (e.g., Barsalou, 2016). While for EG views it is easy to argue that concrete concepts such as “chair” and “cat” are grounded in sensorimotor and emotional systems, it is less straightforward to contend that this is the case for abstract concepts like “justice” and “phantasy.” Concrete concepts typically have a single and clearly bounded referent, while abstract ones do not; furthermore, they are generally more complex, they refer more frequently to complex events or situations (Kiefer and Barsalou, 2013), and they are “progressively more detached from physical experience” (Barsalou, 2003; Fernandino et al., 2015) than concrete concepts, even if evidence has shown that they are also grounded in perceptual modalities, particularly in visual ones (Connell and Lynott, 2012). Concrete concepts are typically processed faster and remembered better than abstract ones (concreteness effect, Schwanenflugel et al., 1988, but see counterevidence by Kousta et al., 2011, and no evidence by Barca et al., 2002), and in feature generation tasks they typically elicit more social aspects of situations and more introspective features (Barsalou and Wiemer-Hastings, 2005). As to their neural underpinnings, abstract concept's processing engages more left-lateralized brain areas like the left inferior frontal gyrus and the left middle temporal lobe (see meta-analysis by Wang et al., 2010) and knowledge on abstract concepts is impaired in syndromes such as deep dyslexia and semantic dementia (Shallice and Cooper, 2013). In spite of behavioral and neuropsychological evidence showing differences between concrete and abstract concepts, it is difficult to contend that they are dichotomously organized, since abstractness and concreteness are graded, and different sub-kinds of concepts exist. We therefore start from the assumption that they are arranged along a continuum spanning from highly concrete to highly abstract concepts. In line with the idea of a continuum, psychological, and neuroscientific studies have recently started to investigate fine-grained distinctions among kinds of abstract concepts, analyzing for example the differences in behavioral effects and neural representation of mental state concepts, social concepts, institutional concepts, mathematic concepts, and emotional concepts (Setti and Caramelli, 2005; Crutch et al., 2013; Ghio et al., 2013, 2016; Roversi et al., 2013). Hence, the category of abstract concepts is highly heterogeneous.

### The peculiarity of emotional concepts

Emotional concepts in particular represent a special case since they have an ambiguous status. From the point of view of an embodied theory, clearly emotional concepts are less difficult to handle with than pure abstract concepts, since when compared with abstract concepts it is much easier to demonstrate that they activate bodily sensations and are grounded in sensorimotor and emotional systems (Borghi and Binkofski, 2014). Empirical research has provided contradictory results. In many studies emotional concepts are considered as a subset of abstract concepts, on the basis of abstractness, concreteness, and imageability ratings provided by participants. Other evidence has instead demonstrated that emotions represent a distinctive kind of concept when compared to both abstract and concrete ones (e.g., Altarriba et al., 1999; Altarriba and Bauer, 2004): they are recalled better than both concrete and abstract words, they are rated differently from both concrete and abstract concepts in concreteness, imageability, and contextual availability, they elicit more different associations, followed by abstract and then by concrete words; finally, independently from their polarity (negative or positive) emotion words are processed faster than other words (Kousta et al., 2009).

As to their development, concrete emotion words are acquired before abstract emotion words. Recent data showed that valenced abstract words are acquired before other abstract words (Kousta et al., 2011, Figure 7, p. 26; Ponari et al., 2017) and that emotional valence facilitates the acquisition of abstract concepts in school-age children (Ponari et al., 2017). Their early acquisition has been related to the later acquisition of abstract concepts by proponents of the Affective Embodiment Account (AEA). According to the AEA, emotional experience dominates representation of abstract words. Consistently, learning of emotional terms provides a bootstrapping mechanism useful to learn abstract concepts (Kousta et al., 2011; Vigliocco et al., 2013). Since emotional concepts are the first concepts to be acquired that do not possess a concrete referent but rather refer to interoceptive states, they can facilitate the acquisition of abstract concepts, which do not have a concrete referent.

In spite of this hypothesis, to the best of our knowledge, no study directly investigates acquisition and representation of concrete, abstract not-emotional and abstract emotional concepts in children starting from a perspective in which the effects of the bodily involvement on acquisition are analyzed (Pexman, 2017).

The first aim of our paper is to investigate how 7-years-olds represent concrete, abstract not-emotional, and abstract emotional concepts, in order to verify whether abstract emotional concepts can be assimilated to other abstract concepts or represent instead a distinctive kind of concepts. We wanted to investigate conceptual development in children who had just started a formal linguistic education at school, i.e., first-graders.

We decided to use a word definition task that is typically used to test lexical access, retrieval of stored lexical information, as well as language production in typically developing children and impaired population (Burani et al., 2006; Caramelli et al., 2006). The word definition task would allow us both to verify whether children are able to provide correct definitions of the word meanings as well as to analyze and directly compare the network of conceptual relations elicited by the three different kinds of concepts. Stimuli were chosen taking also into account written frequency of texts for first-grade children (Marconi et al., 1993). The selected corpus ensures that children of this age and school class had been exposed to the experimental stimuli. Although we are well aware that abstract concepts are rather heterogeneous and that all abstract concepts might be emotionally connoted, we distinguished purely emotional concepts from other abstract concepts, in order to verify whether abstract emotional concepts can be assimilated to abstract concepts or whether they are represented and processed differently from not-emotional abstract concepts.

The second aim of this work is to investigate whether the representation of the three kinds of concepts in 7-years-old children is differently affected by the use of pacifier in the period of the linguistic burst. The reason why we are interested in the long-term effects of the pacifier use is that, according to some embodied cognition theories and evidence on abstract concepts and on emotional development, the mouth activation plays a crucial role for representing abstract and abstract emotional concepts compared to concrete ones. In the following we first explain why we think that the activation of the mouth is critical for abstract concepts representation and processing, and then we overview some studies on pacifier use and explain why we hypothesize that the acquisition of abstract not-emotional and abstract emotional concepts might be influenced by pacifier use.

### Abstract concepts and activation of the mouth

As to abstract concepts and the activation of the mouth, we will here focus on the WAT (Words As social Tools) view (Borghi and Cimatti, 2009; Borghi and Binkofski, 2014), that underlies how the different acquisition modality of concrete and abstract concepts influences their later representation (see also Wauters et al., 2003). According to the WAT view, both sensorimotor and linguistic-social information concur in representing concrete and abstract concepts, but this information is differently distributed. While the experience with the physical environment has a major weight for the acquisition of concrete concepts, the social, and linguistic input provided by others is crucial for acquiring abstract concepts, since they do not possess a single referent, which can be easily identified through the senses. The first grounded view that highlighted the role not only of sensorimotor but also of linguistic information for characterizing concepts is the LASS (Language and Situated Simulation) view, according to which linguistic representation are more superficial while conceptual content resides in situated simulations (Barsalou et al., 2008). While WAT is strongly inspired by the LASS view, it differs from it for at least two reasons: because it focuses on abstract concepts representation; and because it ascribes more relevance to the linguistic experience as a whole and does not consider language only as a shortcut to access to content, which would be represented only in sensorimotor terms (for a more thorough analysis, see Borghi et al., 2017). In the WAT view language experience plays a crucial role: beyond its communicative role, language influences categorization, supports prediction (Lupyan and Clark, 2015) and, more generally, it can be seen as a tool that widely extends our thought capabilities (Dove, 2014) and affords the realization of a human-specific pedagogical context for efficient learning (Csibra and Gergely, 2009; Pezzulo et al., 2014). In the case of abstract concepts, linguistic labels can thus provide a glue useful to put together category members that can be highly diverse and variable; in addition, language can be a means useful to introspectively reason on abstract concepts and to focus on inner states (Barsalou and Wiemer-Hastings, 2005; Kiefer and Pulvermüller, 2012; Kiefer and Barsalou, 2013). In line with this view, it has been shown that abstract concepts, differently from concrete ones, benefit from rich linguistic contexts (Recchia and Jones, 2012) and that, beyond sensorimotor features, they incorporate more linguistic, social and also interoceptive features than concrete concepts (Thill and Twomey, 2016).

According to the WAT proposal, the major role played by language in the representation of abstract concepts has an embodied counterpart: the activation of the mouth (see Topolinski and Strack, 2009). A number of recent studies seem to support the link between abstract concepts and activation of the mouth. We will briefly review this evidence.

fMRI studies have shown that processing of abstract words engages brain areas dedicated to language processing. A recent meta-analysis (Wang et al., 2010) on abstract concepts processing reports involvement of linguistic production and comprehension areas, in particular the left inferior frontal gyrus (Broca's area) and the left middle temporal lobe. Literature has shown that the LIFG is involved in subvocalizations and in phonological processing and working memory, and it has been hypothesized that abstract words remain longer in working memory in phonological form due to their difficulty (Binder et al., 2005). The activation of these “linguistic” areas is thus compatible with the activation of the mouth. Many behavioral studies have confirmed that abstract word processing implies the activation of the mouth. Experiments on adults in which the acquisition of novel categories and words was mimicked, using novel figures or Lego objects, revealed that new abstract words were responded to faster with the microphone, while new concrete words elicited faster responses with the keyboard (Borghi et al., 2011; Granito et al., 2015). Furthermore, two ratings studies with Italian words derived from two different databases (Barca et al., 2002; Della Rosa et al., 2011) confirmed that abstract words were rated higher on involvement of the mouth, concrete ones of the hand (Granito et al., 2015; Borghi and Zarcone, 2016); higher involvement of the mouth was also found in a rating study with abstract and emotional sentences (Ghio et al., 2013). The significant advantage of abstract categories in the ratings on mouth involvement was true also comparing them with concrete categories involving heterogeneous members, thus it did not depend exclusively on the differences between the category members (Granito et al., 2015). A further study with an implicit definition-word matching task (Borghi and Zarcone, 2016) revealed that the advantage in response times of the hand over the mouth responses was more marked with concrete than with abstract concepts. Two possible explanations have been provided, which are not necessarily mutually exclusive. The first is that the activation of the mouth with abstract concepts might depend on the re-enactment of their peculiar acquisition modality, which strongly involves linguistic explanations. The second is that, given the higher complexity of abstract words, we might need to re-explain their meaning to ourselves, possibly through a form of inner talk.

### Effects of pacifier use on acquisition of abstract and abstract emotional words

One way to test whether the mouth involvement is critical during abstract terms acquisition is to investigate the effect of an oral device, as the pacifier, on language development (for further work showing how words referring to emotions, as “to smile,” activate the corresponding facial muscle, see Foroni and Semin, 2009). The debate on the use of the pacifier is currently very lively, but mostly confined to its implications for feeding babies (e.g., negative implications for breastfeeding) or for teething and orthodontic problems. Very little is known as to the cognitive-linguistic and emotional implications of its extensive use. Two recent studies link the use of the pacifier with the emotional competence (Niedenthal et al., 2012; Rychlowska et al., 2014). According to the authors, prolonged use of pacifier (duration and frequency of use) would result in an altered facial expression in children and, subsequently, in a reduction in emotional skills (e.g., expressing emotions through facial expressions, and recognizing emotions expressed in faces of others); the effect occurred only in male babies. In light of the numerous studies that show that in adults the mobility of the facial muscles is important in the development of emotional material (e.g., reduced mobility caused by Botox injection affects the ability to process “faces and emotional words,” Havas et al., 2010), there may be an interaction between pacifier use and early emotional development of the child, where the use of the pacifier for several hours during the day, and in social contexts, induces a particular motility/location of facial muscles (if the pacifier is used only at night or sleep it may have a minor impact). While recent evidence has shown a negative relation between pacifier use and children's emotional competence (Niedenthal et al., 2012; Rychlowska et al., 2014), the possible interaction between use of pacifier and learning and processing of emotional language has not yet been investigated.

Less is known regarding the relationship between pacifier use and abstract concepts acquisition. As anticipated in the introduction, several studies have shown that the role of linguistic and social input is more relevant for the formation of abstract concepts than of concrete ones, and that this leads to an activation of the mouth (for a review see Borghi et al., 2017). As the representation of abstract concepts not only counts on sensorimotor information but also on linguistic and social information, the overuse of pacifier may interfere with the acquisition of abstract concepts. In other words, because pacifiers occupy the mouth, and because abstract words elicit motor simulations of mouth action, then abstract concepts might develop differently in infants who use pacifiers.

### Aims and hypotheses

The present study aims to verify whether the extended use of pacifier interferes with the acquisition and consolidation in memory of abstract not-emotional and abstract emotional words meanings compared to that of concrete ones. Specifically, we intend to investigate long-term effects of pacifier overuse using a definition task with 7-year-olds who have never used the pacifier, who have used it for short (until 2 years), until age 2–3 or beyond age 3.

Based on the aforementioned review, we formulate two hypotheses. The first pertains the distinction between the three conceptual kinds (abstract not-emotional, concrete, and abstract emotional). The second concerns the possible influence of pacifier overuse on the acquisition and consolidation of abstract emotional and not-emotional concepts.

### Distinction between abstract, concrete, and abstract emotional concepts

We contrast two possible views. According to the first, emotional concepts can be considered as a subset of abstract concepts (e.g., Kousta et al., 2011). If this is the case, then we should find neither differences in accuracy between abstract emotional and not-emotional concepts nor differences between the network of relations elicited by them; both emotional and abstract concepts should differ from concrete concepts. According to the second view, emotional concepts do not represent a subset of abstract ones but rather differ from both concrete and abstract concepts (Altarriba et al., 1999; Altarriba and Bauer, 2004; Setti and Caramelli, 2005); consistently, abstractness and valence have different neural representation (Skipper and Olson, 2014). If this is the case, then the conceptual relations characterizing emotional concepts should differ from those elicited by both concrete and abstract not-emotional ones.

### Effects of pacifier use on conceptual acquisition

We predict an influence of the pacifier overuse on the acquisition of both abstract emotional and not-emotional concepts, for which the linguistic and social context of acquisition is particularly important. The use of pacifier would namely render more difficult the formation of a linguistic simulation activating the mouth, and due to its effect on facial expression, it would render social and emotional interactions more difficult. In contrast, the pacifier should not affect the acquisition of concrete concepts, for which the simulation with the mouth would not be necessary and the role of facial expression might be less relevant.

While we predict an influence of the use of pacifier on conceptual development of abstract and emotional concepts, we intend to contrast a stronger and a milder hypothesis. According to the strong hypothesis, pacifier overuse would interfere with the acquisition of abstract and abstract emotional concepts. To test this hypothesis we scored the definitions provided by children distinguishing them in fully correct, partially correct, and incorrect or no response. The strong hypothesis predicts a higher number of incorrect or missing definitions with abstract and emotional concepts than with concrete concepts, particularly in children who used the pacifier beyond age 3.

According to a weak hypothesis, pacifier overuse would influence the organization of conceptual relations elicited by concepts. Accordingly, the three kinds of concepts should not differ in the number of correct definitions but in the pattern of conceptual relations they elicit. To test this hypotheses we coded the definitions provided identifying different kinds of conceptual relations (see section Scoring of the Responses), and we analyzed the pattern of semantic relations characterizing concrete, abstract, and emotional concepts. The weak hypothesis predicts that the pattern of conceptual relations produced by late users of pacifier should be characterized by a less marked difference between concrete and abstract concepts and between concrete and emotional concepts.

## Experiment 1: definition task

### Methods

#### Participants

The sample included 59 children aged 6–7 years (28 male) from a school of Rome. As part of the recruitment procedure, children' parents provided their Informed Consent by means of an enrolling questionnaire requiring information about family composition, socioeconomic status, familiarity with other languages, children cognitive, auditory or language impairments, and pacifier use (if any). Parents also indicated if their child used a pacifier (a) during the day at home, (b) at night, and (c) during the day outside of the home, including school (see also Niedenthal et al., 2012). No a priori selection has been made, that is all children with approved Consent participated in the study.

Data of children who had a language impairment certification were not included in the study.

The demographic characteristics of the sample are reported in the Appendix 2.

Results discussed in the following sections are based on a reduced sample of 46 participants, as in 22% of cases parents filled the questionnaire but did not provide information about pacifier use (in the Appendix, participants not included are marked by an asterisk).

Children were classified into four subgroups based on parents' responses:

– *Never:* Children who never used the pacifier (six participants, three males);
– *Two*: those who used the pacifier up to 2 years of age (17 participants, 10 males);
– *Two-Three:* those who used the pacifier up to Two-Three years of age (18 participants, nine males);
– *Three:* those who used the pacifier for 3 years of age and more (five participants, three males) (Table 1).

**Table 1:** Demographic characteristics (percentages) of participants.

**Pacifier**	**Gender**		**Age in months**	**Schooling mother**				**Schooling father**					**Exposure to other languages**
	**Male**	**Female**	**Mean (range)**	**Middle school**	**High school**	**University**	**NA**	**Elementary school**	**Middle school**	**High school**	**University**	**NA**	
Never	50	50	78.3 (73–83)	17	50	33	0	17	17	50	17	0	50
Two	59	41	77.1 (69–83)	12	41	41	6	0	12	59	18	12	24
Two-Three	50	50	77.5 (69-83)	22	56	17	6	6	22	61	6	6	22
Three	60	40	77.2 (72–80)	40	20	40	0	0	20	20	60	0	20

Overall, the distribution of pacifier use was not homogenous, with most of the children in our sample who have used it (87%). A large proportion of them used the pacifier for sleeping purposes (86% during the night, 60% during daytime at home), in few cases they used it also at home (33%) or at school (27%), presumably during social interaction but we do not have further information. The school is located in a multiethnic suburb of Rome, thus 26% of the children had been exposed to other languages, such as English, Singhalese, Portuguese, Spanish, German, Moldovan, Pakistani, Arabic, Albanian, and Romanian. Chi square analyses revealed that the demographic information did not significantly differ between the pacifier groups (Chi-square = 12, *df* = 9, *p* > 0.1).

#### Materials and design

A list of 30 Italian words (10 abstract, 10 concrete, and 10 emotional words) was selected from a larger sample explored in a preliminary study (see Supplementary Materials, Appendix 1). Stimuli characteristics are presented in Table 2. As it can be seen in the appendix, all emotional terms we selected refer to basic emotions (e.g., *fear*) or to emotional states (e.g., *love*) or are concepts that for children are strongly associated to possession and transmission of emotions (e.g., *kiss, heart*) (Table 2).

**Table 2:** Psycholinguistic characteristics of the stimuli used for the definition task.

	**ABS**	**CONC**	**EMO**	**FREQ**	**IMA**	**FAM**	**AoA**	**MoA**	**Length**
**Abstract words**	432 (69)	305 (71)	3.2 (1.1)	89 (104)	390 (141)	534 (82)	326 (70)	46 (81)	7.2 (0.8)
**Concrete words**	124 (30)	676 (27)	2.0 (0.6)	28 (13)	668 (17)	562 (75)	251 (48)	226 (73)	7.3 (1.8)
**Emotional words**	410 (128)	367 (148)	5.7 (0.3)	113 (173)	445 (117)	595 (63)	267 (70)	369 (96)	6.2 (1.6)

*ABS, abstractness; CONC, concreteness; EMO, words emotionality; FREQ, written word frequency; IMA, imageability; FAM, familiarity; AoA, word age of acquisition; MoA, concept mode of acquisition; Length, word length in letters. Mean values and standard deviation are provided (in bracket)*.

Attention was made in order to control for correlated variables. Nevertheless, abstract words had lower values of Concreteness, Imageability, AOA, Context Availability and MoA than concrete words; concrete words had lower values of Abstractness, Imageability, AOA, Context Availability and MoA than emotional words; and Emotional words had higher values of MoA than abstract words (*p*s < 0.05, *t*-test computed in Excel). Importantly, the emotional words we selected had abstractness values only slightly lower than abstract concepts and concreteness values much lower than concrete concepts (see Table 2), thus they can be considered, according to the ratings, as subsets of abstract concepts, even if they differed in the acquisition modality, which was mainly linguistic for abstract concepts. Aside from acquisition modality, the main difference between the selected abstract and emotional concepts concerns their valence. To be certain that abstract concepts and emotional concepts differed in emotionality, we performed paired sample *t*-tests (Bonferroni corrected) on the ratings obtained in order to test whether there was a significant difference in emotional ratings between emotional, abstract, and concrete concepts. Even if abstract words were considered as more emotional than concrete ones [*t*_(9)_ = 3.05, *p* = 0.014], emotional words (*M* = 5.67) were evaluated as significantly more emotional than both concrete words (*M* = 2.04), [*t*_(9)_ = 15.11, *p* = 0.001], and other abstract words (*M* = 3.16) [*t*_(9)_ = 7.01, *p* = 0.001], confirming our expectations. Crucially, no abstract word was evaluated higher than any emotional word in emotional valence (see Supplementary Table 1 and Supplementary Figure 1).

#### Procedure

Children were enrolled directly at school where data acquisition took place. They were picked up individually from the class and taken to a room specifically dedicated to data collection. They were asked to sit at the table with the experimenter, and a plastic bowl containing pieces of paper was put in front of them. They were asked to pick up a piece of paper one at a time and to provide an oral definition of the word that the experimenter read to them. All the responses were typed online on the computer and were audio recorded. Each session lasted between 15–20 min and, at the end of the session, the child was taken back to the classroom.

#### Scoring of the responses

Definitions were rated using two scoring systems. *Level 1* pertained the accuracy of the response and used a three point scale (2 = fully correct, 1 = partially correct, 0 = not correct or no response, see also Burani et al., 2006).

*Level 2* focused on the qualitative analysis of the response, using 11 categories based on the conceptual relations elicited in the response. We assigned one point to each category. Categories Definitions' Features scoring system were:

1. *Perceptual features* (referring to perceptual properties of the concept, e.g., “helicopter: something that has a propeller”);
2. *Thematic-Spatial* (referring to spatial location, e.g., “library: where the books are”);
3. *Thematic-Action-Function* (referring to the functionality of the concept, e.g., “box: you put something inside”);
4. *Emotion* (using emotional terms, e.g., “heart: something that is inside us and makes us kind”);
5. *Situation* (referring to situation and events when the concept might occur, e.g., “shame: when you ashamed to do a play”);
6. *Experiential* (referring to some experiences, e.g., “brush: when the teacher tells me to paint something and I paint with the brush”);
7. *Interaction* (referring to social-interactive situation, e.g., “surprise: when someone gives you something and you do not know what it is”);
8. *Taxonomic-Superordinate* (referring to the a higher level of taxonomy, e.g., “banana: it's a fruit”);
9. *Taxonomic-Subordinate* (using an example to define the concept, e.g., “agreement: when you get along with a friend”);
10. *Norm* (referring to social norms, e.g., “helmet: you have to put it on your head when you ride a motorcycle”);
11. *Free Association* (free association with no conceptual relation with the concept, e.g., “culture: when in the morning you have to go to school and have to wear an apron”).

The scoring system we selected was based on previous literature on conceptual development and conceptual representation: in addition to the perceptual/property relations and to the thematic (spatial, action-function, and situation/event) and taxonomic relations (Borghi and Caramelli, 2003; Kalénine et al., 2009; Estes et al., 2011; Mirman et al., 2017), we added free associations, which according to Barsalou and Wiemer-Hastings (2005) should be more typical of abstract concepts, and normative relations, which might characterize abstract concepts of the normative kind (see Roversi et al., 2013). Finally, since we were interested in the role of direct experience and of emotional and social aspects in characterizing abstract concepts we added experiential, emotion (see also Wu and Barsalou, 2009), and interaction relations.

#### Reliability analysis

Two independent coders (the first two authors of the study) used the two level systems to rate the definitions. A third coder (the last author) intervened in case of disagreement. Inter-judge reliability of coding was calculated by means on inter-rated *t*-test, which showed no significant difference (*t*-value < 1).

### Data analysis and results

The results were first analyzed considering overall accuracy (total correct definitions). *Generalized linear mixed-effects model* (GLMM) was used to assess the impact of Concept type and Pacifier use on accuracy data (Baayen, 2008; Bolker et al., 2009). Second, a qualitative analysis of conceptual features was conducted, focusing on conceptual content underlying children's definitions (Borghi and Caramelli, 2003; Caramelli et al., 2006). *Correspondence analysis* (CA) was used to explore relationships among our categorical variables (Concepts type and Pacifier use) and the conceptual relations used in the word definition task. CA is a statistical exploratory technique used to graphically visualize the underlying structure of contingency table. *Hierarchical clustering* performs an agglomerative hierarchical grouping on results of the CA.

#### Accuracy of definitions

**Figure 1:**
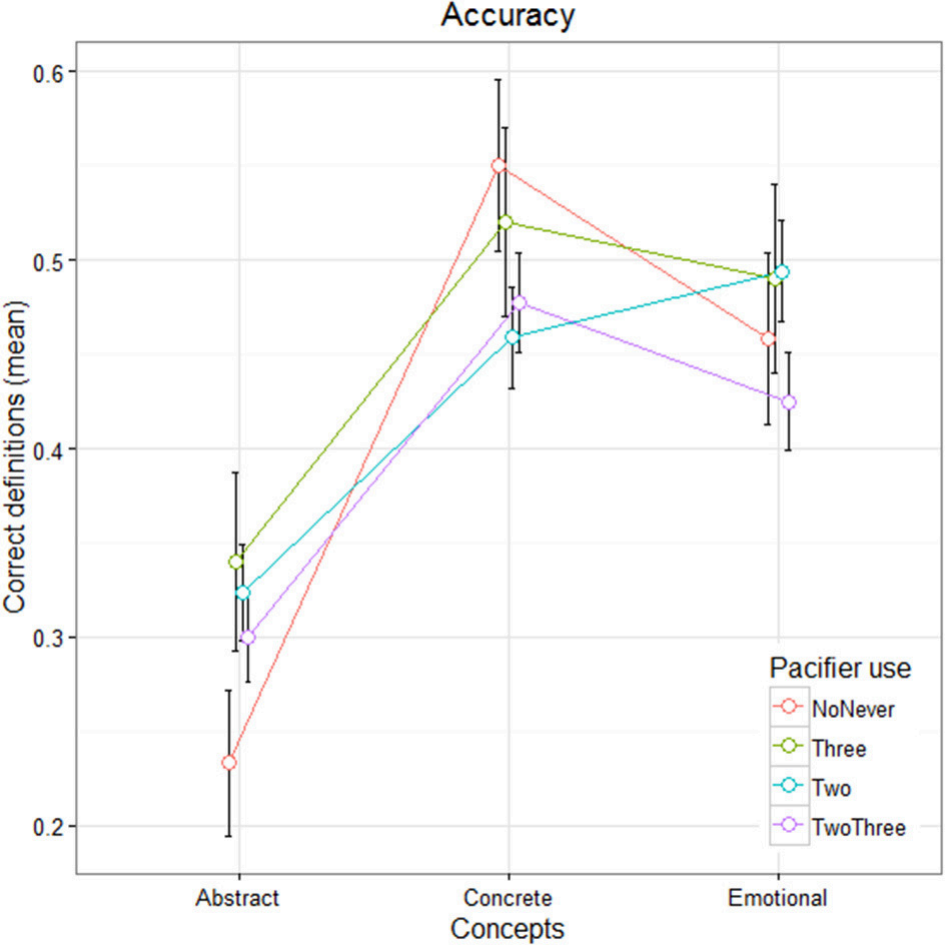
Line graph showing mean correct definitions as a function of Concept type and Pacifier use. Error bars indicate standard error means.

Overall children were accurate in completing the definition task, with 89% of correct response (44% of the total were considered fully correct, 45% of the total partially correct), 10% of errors and a small percentage of no responses. Correct definitions as a function of Concept types and participants' group are presented in Figure 1.

Generalized linear mixed model (GLMM) fit by maximum likelihood (Laplace Approximation) was used to assess the impact of Concept type and Pacifier use on accuracy data. GLMM was implemented in R Development Core Team (2005) with the lme4 package, with parameter “family = binomial” to account for categorical data (Bates and Maechler, 2009). The model included random intercept for Subjects and Items, and fixed effect of Concept type (Abstract, Concrete, and Emotional) and frequency of Pacifier use (Never, up to 2 years, Two-Three years, more than 3 years). Following the recommendations of Barr et al. (2013), we also included by-subject random slope in the model (that is introducing by-subject adjustments to the intercept as well as by-subject adjustment to the fixed factor Concept).

As the data distribution presents many zeros, we used the Akaike's Information Parameter (AIC) to evaluate the most suitable model to data analysis^1^. Models comparison showed that the LMM is better (*df* = 20, AIC = 3,135) than the Poisson logit hurdle model (PLH: Log L = −1832, *df* = 24, AIC = 3,712) and the zero-inflated negative binomial logit hurdle model (NBLH: Log L = −1,832, *df* = 25, AIC = 3,713).

GLMM model showed significant differences between Abstract and Concrete concepts (β_Concrete_ = 2.27, *z* = 3.25, *p* < 0.001), and between Abstract and Emotional concepts (β_Emotional_ = 1.6, *z* = 2.6, *p* < 0.001). No differences emerged between Concrete and Emotional concepts (β_Concrete:Emotional_ = −0.47, *z* = −0.89, ns), nor between different frequencies of pacifier use (*z* < 1). Neither the Concept type per Pacifier interaction was significant, except for the contrast Concrete vs. Pacifier Two (β_Concrete:PacifierTwo_ = −1.4, *z* = −2.133, *p* < 0.05)^2^. Abstract concepts were more difficult to define than both Concrete and Emotional ones and, more interestingly, no differences emerged between Concrete and Emotional concepts, as shown also in Figure 1.

#### Conceptual content of definitions

Correspondence analysis (CA) was used to explore relationships among our categorical variables (Concepts type and Pacifier use) and the conceptual relations used in the word definition task, which we named “Definitions” Features' (see also Caramelli et al., 2006; Sourial et al., 2010; Ghio et al., 2013). The logic underlying Correspondence analysis is quite similar to that of principal component analysis, but CA applies to categorical data. In CA the frequencies of the conceptual relations give rise to a two-dimensional graphical form where they are represented as points in a multidimensional space. The geometrical proximity of the points on the graphs indicates the degree of their association and the similarity of their distribution (Greenacre and Blasius, 1994; Greenacre, 2007). The distances between the points are the weighted distances (Chi-square) between the relative frequencies and not the simple Euclidean distances. Correspondence analysis can be also considered as a method for decomposing the overall *Chi-square* statistic by identifying a small number of dimensions in which the deviations from the expected values can be represented. In the analyses we conducted we will have two dimensions, the first of which explains the higher amount of inertia. Specifically, we used the “CAinterprTools” package implemented in R statistical environment (Alberti, 2015).

**Figure 2:**
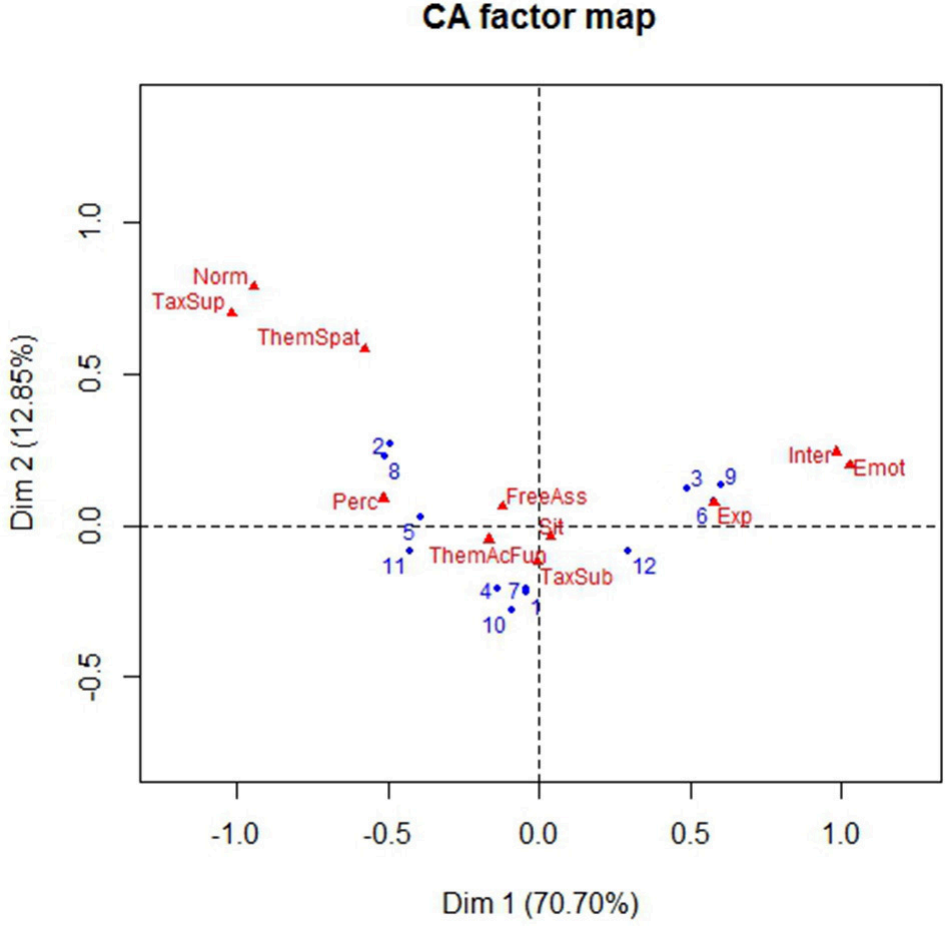
Correspondence Analysis scatterplot of the sub-space defined by dimension 1 and dimension 2. In red: Exp, Experience; Inter, Interaction; Emot, Emotion; Perc, Perceptual; TaxSup, Taxonomic-Superordinate; Norm, ThemSpat, Thematic-Spatial; FreeAss, Free Association; ThemAcFun, Thematic-Action-Function; TaxSub, Taxonomic-Subordinate; Sit, Situation. In blue: 1, Two-Abstract; 2, Two-Concrete; 3, Two-Emotional; 4, Two-Three-Abstact; 5, Two-Three-Concrete; 6, Two-Three-Emotional; 7, Never-Abstract; 8, Never-Concrete; 9, Never-Emotional; 10, Three-Abstract; 11, Three-Concrete; 12, Three-Emotional.

Among the 10 dimensions emerged from the CA analysis, only the first two accounted for most of the inertia, according to the Malinvaud's test (Dimension 1: Eigenvalue = 1.71e+05; Chi-square = 4.96e+08, *df* = 110, *p* < 0.001; Dimension 2: Eigenvalue = 3.11e+04; Chi-square = 1.45e+08, *df* = 90, *p* < 0.001). The scatterplot of the first two CA dimension is in Figure 2.

**Figure 3:**
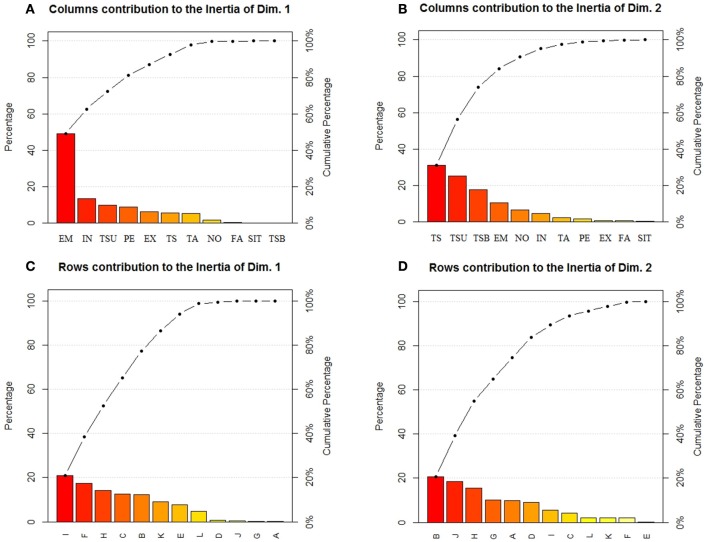
ParetoChart showing which category of Definitions' Features have a higher (in relative terms) contribution to Dimension 1 **(A)** and Dimension 2 **(B)**. Contribution of Pacifier and Concepts type to the definition of Dimension 1 **(C)** and Dimension 2 **(D)**. A, TwoAbstract; B, TwoConcrete; C, TwoEmotional; D, Two-ThreeAbstract; E, Two-ThreeConcrete; F, Two-ThreeEmotional; G, NeverAbstract; H, NeverConcrete; I, NeverEmotional; J, ThreeAbstract; K, ThreeConcrete; L, ThreeEmotional.

To make the results more understandable, Figure 3 shows ParetoCharts with the contribution of different variables to the definition of the first two dimensions of the Correspondence Analysis. The charts in Figures 3A,B show the contribution of Definitions' Features to dimension 1 and 2 respectively. Different types of conceptual relationships are contributing to the determination of the two dimensions. In correspondence analysis the first dimension is typically more important than the second. Emotion, Interaction type of definitions have a higher contribution on dimension 1 (with a percentage of explained inertia of 49 and 13.4%, respectively). Thematic-Spatial, Taxonomic-Superordinate, and Taxonomic-Subordinate conceptual relations have a higher contribution on dimension 2 (with a percentage of explained inertia of 31, 25, and 18%, respectively).

Figures 3C,D shows the contribution of Pacifier by Concepts type to the definition of dimension 1 and 2. The 12 levels of Pacifier by Concepts type combination are differently contributing to the definition of the two dimensions. Never used/Emotional concept, Two-Three years/Emotional, Never used/Concrete, Up to 2 years/Emotional and Up to 2 years/Concrete concepts have a higher contribution on dimension 1 (with a percentage of explained inertia of 21, 17, 14, 13 and 14%, respectively). Up to 2 years/Concrete, 3 years and more/Abstract, Never used/Concrete and Never used/Abstract concept have a higher contribution on dimension 2 (with a percentage of explained inertia of 21, 18, 16, and 10%, respectively).

Summarizing, we can see that Dimension 1 (70.70% of the overall variance) is characterized by the opposition between Emotional and Concrete concepts. Emotional concepts are characterized by the presence of Emotion, Interactive, and Experiential relations in the definitions of all children (i.e., Never Used, Up to 2 years, Two-Three years) apart from late-users of pacifier (3 years and more). Concrete concepts are characterized by the presence of Taxonomic-Superordinate, Perceptual, and Thematic relations (both Thematic-Spatial and Thematic Action-Function) in the definitions of children who Never Used the pacifier or stopped early to use it (Up to 2 years). This suggests that children who used less the pacifier distinguish more markedly between Emotional and Concrete terms, and that definitions of Emotional concepts of late users of pacifiers (3 years and more) are less clearly characterized than those of children who used it less.

On the less relevant Dimension 2 (12.85% of inertia) Concrete concepts are characterized by children who Never Used the pacifier or stopped early to use it (Up to 2 years) by Taxonomic-Superordinate and Thematic-Spatial relations; they oppose to Abstract concepts characterized in children who Never Used the pacifier and by late users of pacifiers (3 years and more) by Taxonomic-Subordinate/exemplifications relations. Interestingly, the distinction between concrete and abstract concepts is more marked for children who Never Used the pacifier, followed by children who used it until 2 years of age. As to late users of pacifier (3 years and more), similarly to children who did not use pacifiers they produced exemplifications with abstract concepts, but they do not seem to elicit markedly different relations with concrete and abstract concepts.

As to the distinction between concept kinds, we can notice that Emotional concepts oppose to Concrete concepts on Dimension 1, which explains a higher percentage of inertia, while Dimension 2 is characterized by the opposition between Abstract and Concrete concepts. The results therefore indicate that Emotional concepts represent a specific subset of concepts, which however differ more from concrete than from abstract ones.

Hierarchical clustering has been applied over the CA solution depicted in Figure 3, allowing delineating the structure underlying the dataset by means of “tree” and “clusters” (Husson et al., 2010). The purpose of such analysis is seeking structure in the relations among cases characterized by a number of variables: cases that are similar to each other, in terms of their values for a number of variables, are grouped together forming a cluster.

**Figure 4:**
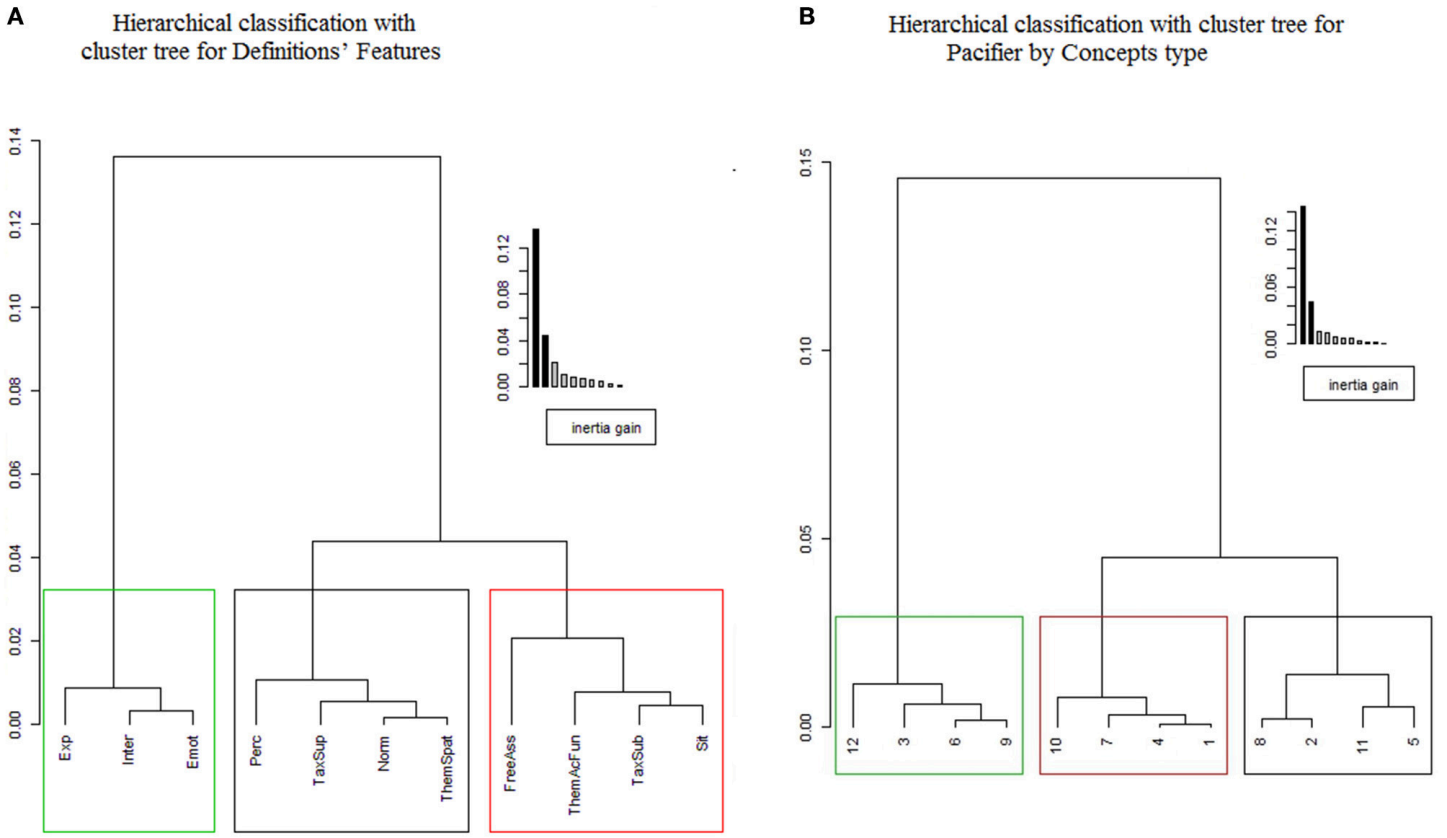
Hierarchical clustering: hierarchical classification with cluster tree for Definitions' Features **(A)** and Pacifier by Concepts type **(B)**. **(A)** *Cluster 3*–Exp, Experience; Inter, Interaction; Emot, Emotion; *Cluster 2*-Perc, Perceptual; TaxSup, Taxonomic-Superordinate; Norm, ThemSpat, Thematic-Spatial; *Cluster 1*-FreeAss, Free Association; ThemActFun, Thematic-Action-Function; TaxSub, Taxonomic-Subordinate; Sit, Situation. **(B)** *Cluster 3*–12, Three-Emotional; 3, Two-Emotional; 6, Two-Three-Emotional*; Cluster 2*–9, Never-Emotional; 10, Three-Abstract; 7, Never-Abstract; 4, Two-Three-Abstract; 1, Two-Abstract; *Cluster 1*–8, Never-Concrete; 2, Two-Concrete; 11, Three-Concrete; 5, Two-Three-Concrete.

Hierarchical clustering is performed using an extension of the “CAinterprTools,” accomplished via the “FactoMineR” Package (Le et al., 2008). The hierarchy is represented by a dendrogram which is indexed by the gain of within-inertia, with optimal level of division between clusters tree indicated by colored boxes. A barplot of the gained inertia is also returned. Hierarchical clustering of the factor map applied to the Definitions' Features (see Figure 4A) resulted in three clusters: Cluster 1 (black square) with Perceptual, Taxonomic-Superordinate, Norm, and Thematic-Spatial relations; Cluster 2 (red square) with Free Association, Thematic-Action-Function, Taxonomic-Subordinate, and Situation; and Cluster 3 (green square) with Experience, Emotion, and Interaction. Cluster 1 and 3 are opposing on Dimension 1, Cluster 1 and 2 are opposing on Dimension 2. The first dimension accounts for the majority of the inertia of the data, and is determined by the opposition between relational features which typically characterize Concrete concepts in opposition to relations typically associated to Emotional concepts. The second dimension is defined by relational features which typically characterize Concrete concepts, in opposition to relations generally associated to Abstract concepts.

The hierarchical clustering of the factor map confirms that Emotional concepts are clearly different from both Concrete and Abstract concepts, but that the major opposition is that between Concrete and Emotional concepts. This opposition clearly does not depend on the level of abstractness, but seem to be due to the fact that the relations evoked by Emotional concepts pertain emotions and interactive situations, with scarce overlap in particular with the relations elicited by Concrete concepts.

As to Pacifier use by Concepts type (Figure 4B), the clusters are characterized as follows: Cluster 1 (black square) with Concrete concepts and different ages of Pacifier use; Cluster 2 (red square) by Abstract concepts and different ages of Pacifier use; and Cluster 3 (green square) by Emotional concepts and different ages of Pacifier use. Cluster 1 and 3 are opposing on Dimension 1, Cluster 1 and 2 are opposing on Dimension 2. Thus the first dimension, which is accounting for the major part of the inertia of the data, is determined by the opposition of Concrete and Emotional concepts. The second dimension, which is defined by Concrete and Abstract concepts, is opposing the former to the latter category. When considering the smaller clusters, for both emotion and abstract concepts late users of pacifiers differ from other children. This seems to confirm our hypothesis that the late use of pacifier influences the development of abstract and emotional concepts.

Subsequent Correspondence Analyses performed separately on Concept type and Frequency of Pacifier use provide a more analytical perspective.

#### Analysis on concept type

Overall, as the percentages show, children extensively uses Thematic-Action-Function and Taxonomic-Subordinate conceptual relations to provide definitions (see Table 3A). This is in line with the predictions of embodied and grounded views, since they seem to situate concepts in action situations and to use exemplifications in order to ground concepts. As to the differences between the concepts, the results of Chi square tests are reported in Table 3B. In line with previous literature, concrete concepts activate more Perceptual properties than Emotional concepts and more Thematic (Thematic-action-function and Thematic-spatial) and Taxonomic-Superordinate relations compared to both Emotional and Abstract concepts. Emotional concepts elicit instead more emotional and more interactive relations compared to Concrete and Abstract concepts, and slightly more experiential relations than concrete concepts (*p* = 0.06). As to Abstract concepts, they are not characterized by any specific kind of relation, even if they evoke more emotion relations than concrete concepts (but less than Emotional concepts) and even if, when we look at the percentages, they seem to elicit a high number of free associations (see for consistent results Barsalou and Wiemer-Hastings, 2005).

**Table 3A:** Percentage of relation and concept type.

	**Perc**	**ThemSpat**	**ThemAcFun**	**Emot**	**Sit**	**Exp**	**Inter**	**TaxSup**	**TaxSub**	**Norm**	**FreeAss**
Abstract	8.04	1.41	43.91	5.11	4.67	3.26	1.96	0.22	59.13	0.22	8.59
Concrete	15.54	11.41	67.50	0.22	5.54	2.83	0.43	6.96	60.54	1.74	5.43
Emotional	3.80	3.15	45.22	35.00	5.98	9.24	11.41	0.33	66.74	0.11	5.54

Perc, Perceptual; ThemSpat, Thematic-Spatial; ThemAcFun, Thematic-Action-Function; Emot, Emotion; Sit, Situation; Exp, Experience; Inter, Interaction; TaxSup, Taxonomic-Superordinate; TaxSub, Taxonomic-Subordinate Norm; FreeAss, Free Association.

**Table 3B:** Chi-squared tests (*df* = 1) on Conceptual relations and Concept kind, Bonferroni-corrected (α < 0.025).

	**Perc**	**ThemSpat**	**ThemAcFun**	**Emot**	**Sit**	**Exp**	**Inter**	**TaxSup**	**TaxSub**	**Norm**	**FreeAss**
Abstract-Concrete	ChiSq = 2.38, ns	ChiSq = 7.80, *p* = 0.005	ChiSq = 4.99, ns	ChiSq = 4.49, ns	ChiSq < 1, ns	ChiSq < 1, ns	ChiSq < 1, ns	ChiSq = 6.33, *p* = 0.01	ChiSq < 1, ns	ChiSq = 1.18, ns	ChiSq < 1, ns
Concrete-Emotional	ChiSq = 7.13, *P* = 0.008	ChiSq = 4.69, ns	ChiSq = 4.40, ns	ChiSq = 34.36, *p* = 0.001	ChiSq < 1, ns	ChiSq = 3.40, *p* = 0.06	ChiSq = 10.18, *p* = 0.001	ChiSq 6.03, *p* = 0.01	ChiSq < 1, ns	ChiSq = 1.44, ns	ChiSq < 1, ns
Abstract-Emotional	ChiSq = 1.52, ns	ChiSq < 1, ns	ChiSq < 1, ns	ChiSq = 22.27, *p* = 0.001	ChiSq < 1, ns	ChiSq = 2.86, ns	ChiSq = 6.68, *p* = 0.01	ChiSq < 1, ns	ChiSq < 1, ns	ChiSq < 1, ns	ChiSq < 1, ns

**Figure 5:**
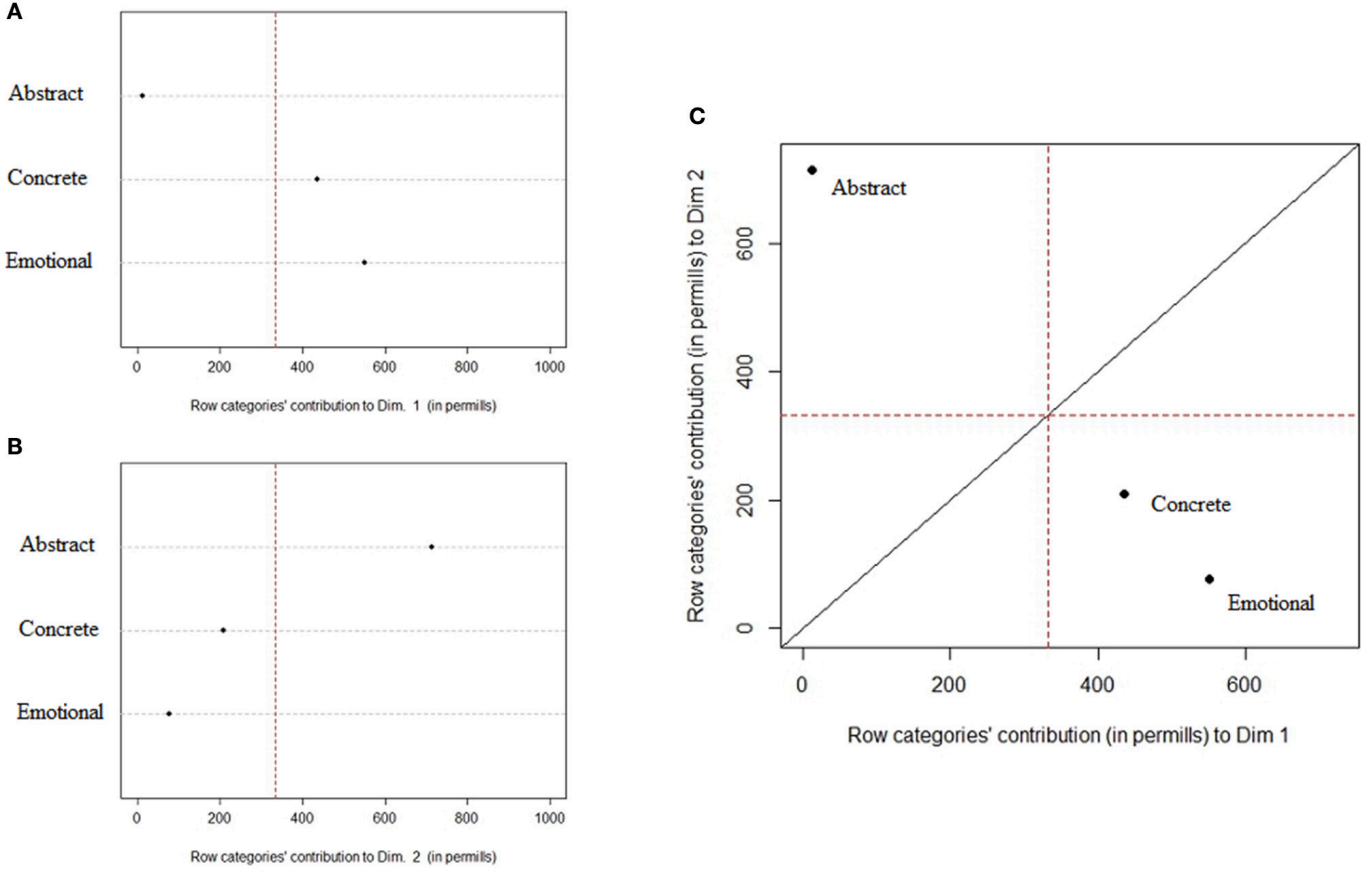
Contribution of Concepts type to the definition of dimension 1 **(A)** and 2 **(B)**; **(C)** Scatterplot of Concepts type's contribution to dimension 1 and 2.

Correspondence Analysis focused on Concept type and the conceptual relations used in the definition task resulted in two dimensions (Dimension 1—Eigenvalues: 0.19, 85.4% of inertia; Dimension 2—Eigenvalues: 0.029, 14.6% of inertia). The charts in Figure 5 highlight which type of concepts is defining the first two CA dimensions. The reference line helps in locating which category has an important contribution to the determination of the dimension.

Concrete and Emotional concepts contribute to the definition of the first dimension, while Abstract concepts provide a major contribution to dimension 2, in line with previous analysis. Differently from the previous analysis, however, Concrete and Emotional concepts can be considered as more similar than Abstract concepts (see Figure 5C).

**Figure 6:**
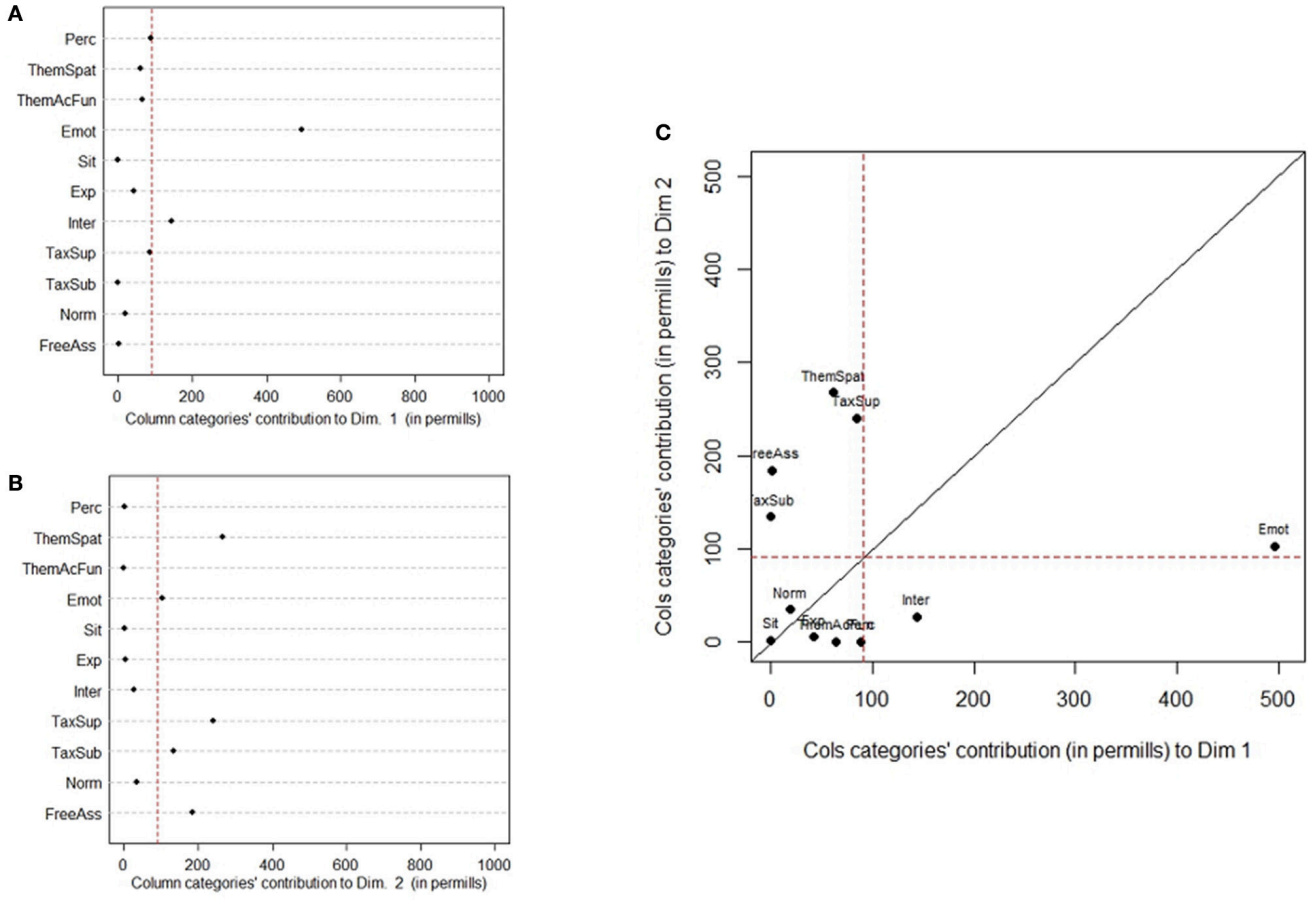
Contribution of the conceptual relations to the definition of dimension 1 **(A)** and 2 **(B)**; **(C)** Scatterplot of conceptual relations' contribution to dimension 1 and 2.

As to the conceptual relations (see Figure 6), Emotional and Interactional type of definitions characterize the first dimension, whereas Thematic-Spatial, Taxonomic-Superordinate, Free Association, Taxonomic-Subordinate and, to a minor extent, Emotional definitions characterize the second dimension.

#### Analysis on pacifier use

Overall, children extensively use Thematic-Action-Function and Taxonomic-Subordinate relations to provide definitions, independently from the frequency of pacifier use (see Table 4A). However, the frequency of these and other relations is modulated by pacifier use.

**Table 4A:** Percentage of conceptual relations and frequency of Pacifier use.

	**Perc**	**ThemSpat**	**ThemAcFun**	**Emot**	**Sit**	**Exp**	**Inter**	**TaxSup**	**TaxSub**	**Norm**	**FreeAss**
Never	11.11	5.28	56.39	17.22	6.94	7.78	3.89	3.61	60.00	0.56	8.33
Two	8.73	6.57	49.90	12.65	5.29	3.92	5.88	2.84	56.96	0.69	9.61
Two-Three	8.43	4.91	49.81	13.61	5.19	5.65	4.26	1.76	65.28	0.93	4.63
Three	10.67	2.67	63.67	11.00	4.67	4.00	2.33	2.67	71.00	0.00	0.67

Perc, Perceptual; ThemSpat, Thematic-Spatial; ThemAcFun, Thematic-Action-Function; Emot, Emotion; Sit, Situation; Exp, Experience; Inter, Interaction; TaxSup, Taxonomic-Superordinate; TaxSub, Taxonomic-Subordinate; Norm; FreeAss, Free Association.

If we look at the percentages, we can see that children who Never Used the pacifier produce a higher percentage of perceptual properties as well as of emotion, situation, and experiential relations; furthermore they produce a high percentage of free associations (but slightly lower than children who used it until 2 years). Children who used the pacifiers until age 2 produce more free associations, more interactive properties and more Thematic-spatial relations than other children. The production of children who used the pacifier until 2–3 years is not characterized by a higher percentage of a specific kind of relations than other groups. Compared to other children, late users of pacifier (3 years and more), produce more properties related to the interaction with the conceptual referents and their perceptual properties, as the higher percentage of Thematic-Action-Function relations and the high percentage of Perceptual relations (but slightly lower than that of children who Never Used the pacifier) testify. Furthermore, they apparently need to ground and exemplify concepts, as the high percentage of Taxonomic-Subordinate indicates.

Overall, the pattern of the relations produced if we look at the percentages suggests that children who did not use pacifier for long produce more relations referring to social and emotional aspects, to experiences and situations, and more free associations, while late users of pacifiers produce mainly exemplifications. If we look at the Chi-squared tests (see Table 4B), we can see however that the only significant differences concern the higher production of free associations of early users of pacifier (Never Used and 2 years) compared to late users (3 years and more). Importantly, free associations are produced more frequently with abstract than with emotional and concrete concepts (see Table 4A).

**Table 4B:** Chi-Squared on conceptual relations and frequency of Pacifier use, Bonferroni-corrected (α < 0.025).

	**Perc**	**ThemSpat**	**ThemAcFun**	**Emot**	**Sit**	**Exp**	Inter^*^	TaxSup^*^	**TaxSub**	Norm^*^	**FreeAss**
Never-2	ChiSq < 1, ns	ChiSq < 1, ns	ChiSq < 1, ns	ChiSq < 1, ns	ChiSq < 1, ns	ChiSq = 1.27, ns	ChiSq < 1, ns	ChiSq < 1, ns	ChiSq < 1, ns	ChiSq < 1, ns	ChiSq < 1, ns
2-2/3	ChiSq < 1, ns	ChiSq < 1, ns	ChiSq < 1, ns	ChiSq < 1, ns	ChiSq < 1, ns	ChiSq < 1, ns	ChiSq < 1, ns	ChiSq < 1, ns	ChiSq < 1, ns	ChiSq < 1, ns	ChiSq = 1.74, ns
2/3-3	ChiSq < 1, ns	ChiSq < 1, ns	ChiSq = 1.69, ns	ChiSq < 1, ns	ChiSq < 1, ns	ChiSq < 1, ns	ChiSq < 1, ns	ChiSq < 1, ns	ChiSq < 1, ns	ChiSq < 1, ns	ChiSq = 2.96, ns
Never-2/3	ChiSq < 1, ns	ChiSq < 1, ns	ChiSq < 1, ns	ChiSq < 1, ns	ChiSq < 1, ns	ChiSq < 1, ns	ChiSq < 1, ns	ChiSq < 1, ns	ChiSq < 1, ns	ChiSq < 1, ns	ChiSq = 1.06, ns
2-3	ChiSq < 1, ns	ChiSq = 1.65, ns	ChiSq = 1.67, ns	ChiSq < 1, ns	ChiSq < 1, ns	ChiSq < 1, ns	ChiSq = 1.53, ns	ChiSq < 1, ns	ChiSq = 1.54, ns	ChiSq < 1, ns	ChiSq = 7.77, *p* = 0.005
Never-3	ChiSq < 1, ns	ChiSq < 1, ns	ChiSq < 1, ns	ChiSq = 1.3, ns	ChiSq < 1, ns	ChiSq = 1.21, ns	ChiSq < 1, ns	ChiSq < 1, ns	ChiSq < 1, ns	ChiSq < 1, ns	ChiSq = 6.52, *p* = 0.01

Correspondence Analysis focused on the frequency of pacifier use and the type of conceptual relations used in the definition task resulted in three dimensions (Dimension 1—Eigenvalues: 0.023, 77% of inertia; Dimension 2—Eigenvalues: 0.004, 14.4% of inertia; Dimension 3—Eigenvalues: 0.003, 9% of inertia).

**Figure 7:**
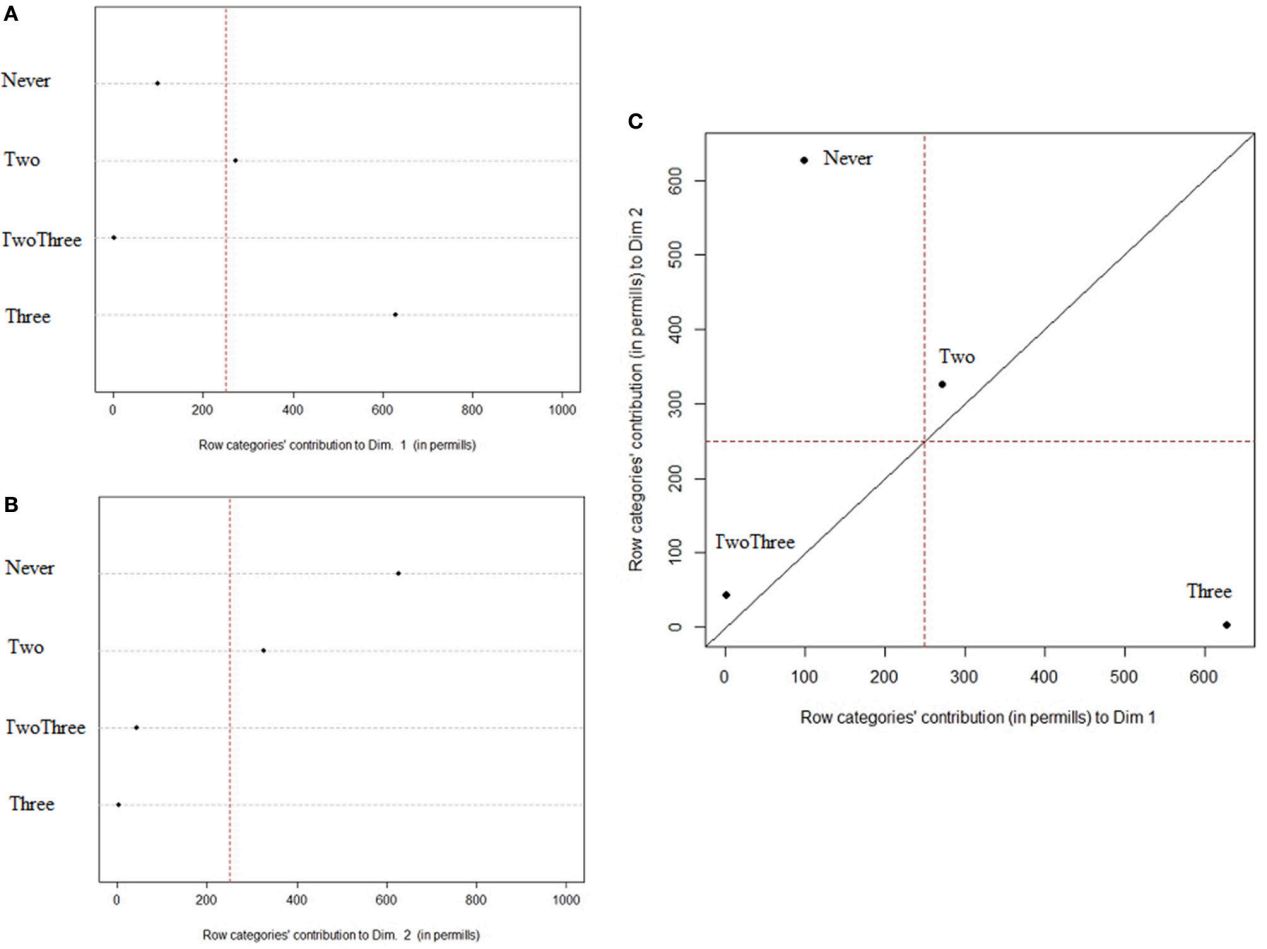
Contribution of Pacifier use to the definition of dimension 1 **(A)** and 2 **(B)**; **(C)** Scatterplot of Pacifier use's contribution to dimension 1 and 2.

The charts in Figure 7 highlight which frequency of pacifier use is defining the first two CA dimension, accounting for the majority of inertia explained.

The first dimension is mainly characterized by the 3-years group, composed by children who used the pacifier beyond age 3, and to a minor extent by the 2-years group. The second dimension is defined by the Never and 2 years groups, that is those who did not use the device or used it for a shorter period. The two Three-years group does not contributes significantly to the first two dimensions. The analysis thus shows that the use of pacifiers has an impact on the pattern of conceptual relations, as indicated by the distinction between late users of pacifiers and those who never used it or stopped to use it early.

**Figure 8:**
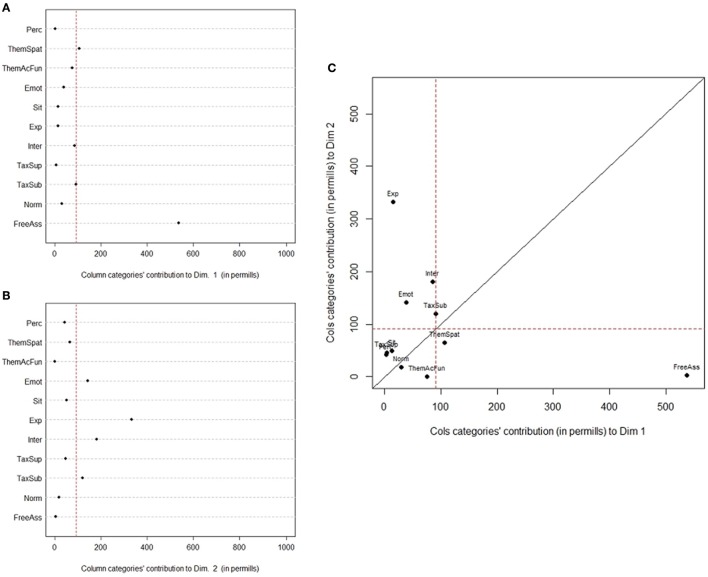
Contribution of conceptual relations to the definition of dimension 1 **(A)** and 2 **(B)**; **(C)** Scatterplot of conceptual relations' contribution to dimension 1 and 2.

As for the conceptual relations (see Figure 8), Free Association and, to a minor extent Thematic-Spatial relations, define the first dimension, whereas the second dimension is defined by Experiential, Interactional, Emotional, and Taxonomic-Subordinate conceptual relations. Interestingly, the use of Free Association is clearly distinct from use of relations more linked to social and emotional aspects, as well as to examples.

## Discussion

### Distinction between abstract, concrete, and emotional concepts

Overall, our results clearly support the studies according to which abstract Emotional concepts differ from other, non-emotional Abstract concepts (Altarriba et al., 1999; Altarriba and Bauer, 2004; Setti and Caramelli, 2005) and are not in line with the view according to which all abstract concepts are emotional ones. As anticipated, abstract concepts come in different varieties, and range from social concepts to mental states to mathematical ones (see Borghi and Binkofski, 2014, for a thorough analysis of this). Here we were interested in the relationship between abstract concepts that directly refer to emotions, i.e., emotional concepts, and other kinds of abstract concepts. Even if emotional concepts are abstract by definition, our results show that abstract Emotional concepts represent a very special sub-kind among the sub-kinds of abstract concepts. Our study widely extends previous results showing that in 7-year-olds abstract Emotional concepts differ from Abstract concepts both in accuracy and in the pattern of conceptual relations they elicit.

As to accuracy, linear mixed-effect modeling showed that Abstract and abstract Emotional concepts differed, due to fact that abstract concepts were more difficult to define than Emotional concepts; Emotional and Concrete concepts instead did not differ.

Emotional concepts differed from both Concrete and Abstract concepts also as to the conceptual relations they yielded. In the correspondence analysis in which the conceptual relations elicited by the three kinds of concepts were combined with contribution of pacifier (Figure 2), Emotional concepts opposed on the first and more relevant dimension to Concrete concepts, while in the correspondence analysis on the conceptual relations yielded by the three kinds of concepts without considering the role of pacifier (Figure 5C), abstract Emotional concepts were represented on Dimension 1 together with Concrete concepts, while Abstract concepts were represented on Dimension 2.

While our results disconfirm views according to which abstract emotional concepts do not differ from abstract non-emotional concepts, they do not exclude that the presence of emotional features might be relevant in characterizing abstract concepts overall, as proposed by a recent view on abstract concepts (Kousta et al., 2011; Newcombe et al., 2012; Vigliocco et al., 2013; Siakaluk et al., 2014). Indeed, results on correlations in the preliminary study (see Figure S1) suggest that emotionality is correlated, even if slightly, with Abstractness, Age of Acquisition and Modality of Acquisition, and show that Context Availability, Concreteness and Imageability are, even if slightly, negatively correlated with Emotionality. Furthermore, Chi squared tests on the conceptual relations produced revealed that abstract concepts yield more emotion relations than Concrete concepts, even if less than Emotional concepts.

The results we found on abstract Emotional concepts have a number of theoretical implications. First, they highlight the limitations of a view according to which Abstract and Concrete concepts are dichotomously opposed, and favor instead the idea that they are arranged along a continuum (Wiemer-Hastings et al., 2001). Indeed, we found clear processing differences between Abstract concepts, Concrete concepts and abstract Emotional concepts. This is particularly interesting because emotional concepts by definition would be part of abstract concepts, since they do not have a concrete object as referent, and also because in our study the selected Emotional concepts had abstractness and concreteness levels similar to Abstract concepts. Second, they suggest that more studies are needed, aimed at investigating the fine-grained differences and the different typologies of concrete and abstract concepts. The fine-grained analysis of differences between kinds of abstract concepts constitutes a new and fruitful research avenue some authors are starting to open (e.g., Crutch et al., 2013; Ghio et al., 2013; Roversi et al., 2013). Third, they indicate that the analysis of conceptual relations elicited might be a promising research avenue to investigate possible differences in the acquisition and development of different kinds of concepts. In spite of the differences we found between Abstract and abstract Emotional concepts, we found one common element: the development of both kinds of concepts is influenced, as predicted, by the use of pacifier.

### Effects of pacifier use on conceptual acquisition

Our results suggest that the use of pacifier has an effect on the development of abstract and emotional concepts. Importantly, this effect is a long-term one, since we tested the conceptual representation in children who do not use pacifier since years. We did not find support for our strong hypothesis that the use of pacifier would influence the accuracy of the produced definitions. However, we found support to our weak hypothesis, according to which the network of associated relations of emotional and abstract concepts differs depending on how long the pacifier was used.

As to the influence of pacifier on abstract Emotional concepts, the correspondence analysis in Figure 2 indicates on Dimension 1 that children who never used or used less the pacifier distinguish more sharply between abstract Emotional and Concrete concepts, and that definitions of abstract Emotional concepts by children who used pacifier beyond age 3 are less clearly characterized than those of children who used it less. The use of pacifier influences also Abstract concepts (see Figure 3, Dimension 2): Children who never used the pacifier and used it until age 2 distinguish more clearly between concrete and abstract concepts, while for late users of pacifier (3 years and more) the distinction between abstract and concrete concepts is unclear. The difference between late users of pacifiers and other children for both emotion and abstract concepts is confirmed by the cluster analysis (Figure 4). If we consider the percentage of conceptual relations produced depending on pacifier use (Table 4), we can notice that the relations produced by late users of pacifiers is confined to exemplifications and thematic-action-function relations, with a reduced richness of relations typically associated to emotional and abstract concepts as experiential, interactive, situational, emotional relations as well as spatial relations and free associations. Chi-squared tests revealed that early and late users of pacifiers differed as the first produce more free associations than the others (see Table 4). Finally, the effects of the use of pacifier on the conceptual relations produced is clearly visible in Figure 7C, where 3 years and more significantly contributed to Dimension 1, while children who never used the pacifier or stopped using it early (Never Used, 2 years) contribute to Dimension 2.

Overall, our results indicate that using pacifiers for long leads to a less marked distinction between concrete and abstract emotional concepts and between concrete and abstract concepts; as to conceptual relations, late users of pacifier tend to refer less to their experience, to social and emotional situations, to use more exemplifications and functional relations, and to use less free associations. One possible limitation of our study resides in the sample size: while the overall sample is composed by 59 children, the two samples of children who did not use the pacifier and of children who used it beyond age 3 might appear small. It should be taken into account, however, that the distribution of our sample reflects the distribution of pacifier use in children: the majority of Italian children use the pacifier since the first month of life (Riva et al., 1999) and the majority of them stops to use it before age 3, slightly before starting nursery school. A further limitation is that children with different levels of pacifier's use were not matched for verbal intelligence and vocabulary size, because we intended to have a general picture of the influence of pacifier on acquisition and representation of different kinds of concepts. This might however reduce the strength of our conclusions, hence further work in which these factors are controlled is needed. Importantly, in our sample the differences in use of pacifier cannot be ascribed to demographic characteristics as gender or level of parental instruction, as seen in Table 1. Further studies will be necessary to understand whether children who use less the pacifier differ from children who use it more on the basis of different variables, as for example the level of activity which might render the use of pacifier more/less necessary. Finally, we did not explore the implications of thumb sucking in the present study. Both pacifier use and thumb sucking influence facial mimicry and mouth muscle position, but such behaviors are quite different from each other. While the use of the pacifier is a “passive behavior” for the child as it is induced by parents, thumb sucking is a child volunteer action that is generally despised by parents as it is considered as a “dirty” action. Moreover, from a practical point of view, thumb sucking is more difficult to control especially because we get this information from retrospective questionnaires compiled by parents. Niedenthal et al. (2012) considered both pacifier use and thumb sucking in their study, and found that (differently from pacifier overuse) thumb sucking has no long-term effects on emotional competence, their variable of interest. The implications of both pacifier use and thumb sucking need to be explored in further studies.

The results we found have a number of implications for current theories of abstract and emotional concepts. In general, the influence of the use of pacifier on conceptual development supports the view that abstract concepts are grounded in sensorimotor experience (e.g., Kiefer and Pulvermüller, 2012), and suggests that both linguistic and social and emotional experience might be important for their acquisition and development (Borghi et al., 2017; Pexman, 2017; Ponari et al., 2017).

As to abstract concepts, they confirm the prediction of the WAT theory according to which the use of pacifier, involving the mouth, should interfere with the acquisition of abstract concepts, and influence the pattern of conceptual relations they elicit. Children who did not use the pacifier or used it for short time seem to be more competent in processing abstract concepts than children who overused the pacifier (for more than 3 years and during social interactions): the pattern of relations the first produce with abstract concepts is richer, and the contrast with the pattern of relations elicited by concrete concepts is more marked and clear in children who did not use the pacifier or used it only for short. An extensive use of pacifier can have impeded them for long time to simulate the word meaning either re-enacting the word acquisition experience and/or re-explaining to themselves their meaning through inner talk. This might have influenced the consolidation in memory of abstract concepts meanings. Importantly, the activation of the mouth revealed by the influence of the pacifier is compatible with the views according to which abstract concepts focus attention on internal states (e.g., Barsalou and Wiemer-Hastings, 2005; Kiefer and Barsalou, 2013): introspection could namely occur through inner talk and could involve the recruitment of the mouth motor system (see Borghi and Zarcone, 2016, for developing this issue).

An alternative and not necessarily contrasting explanation ascribes more relevance to the emotional and social dimension: the pacifier might hide more the facial expression, thus it could impede children to fully benefit of the social input necessary for the acquisition of abstract words. The hypothesis that building abstract concepts requires such social input is in keeping with recent evidence on infants (Bergelson and Swingley, 2013) showing that the comprehension of abstract concepts (e.g., “all gone”) emerges at around 10 months and becomes more stable at around 14 months. Around 9–10 months, children improve their ability to follow the gaze of others (Beier and Spelke, 2012), while around 14 months they develop forms of joint attention (Carpenter et al., 1998).

Even if current results do not allow us to fully disentangle between the two explanations, we believe that the first is more plausible in light of the current results. If we look at the percentages of relations produced (Table 4), we can see that abstract concepts elicit a higher percentage of free associations, rather than of relations involving social and emotional aspects.

Two explanations are possible also in the case of emotional concepts; in this case too the two explanations are not necessarily mutually exclusive. The first relates the difficulty in acquisition/consolidation of Emotional concepts with the reduction of children's emotional competence due the fact that the use of pacifier renders is more difficult to manifest and recognize emotions through mimicry and facial expressions (Niedenthal et al., 2012). The high percentage of emotion and interactive relations produced with Emotional concepts renders this explanation highly plausible.

The second relates the difficulty with Emotional concepts to the fact that the pacifier involves the use of the mouth. Some brain imaging and behavioral studies have shown that emotional concepts activate both the mouth and the hand effectors. Moseley et al. (2012) found with fMRI that processing of abstract emotional words, beyond the limbic regions, engages areas of the precentral cortex activated somatotopically by mouth and hand words. Dreyer et al. (2015) showed that patient CA suffering from lesion in the left supplementary motor area was primarily impaired in abstract-emotional word processing, known to be involved in motor planning independently of a specific effector. At the behavioral level, Ghio et al. (2013) used a rating task in which they asked to what extent actions implied by sentences on emotions, mental states or math concepts referred to the leg, arm or mouth. They found that emotion sentences elicited high ratings for both the hand and the mouth, abstract mental state concepts activated more the mouth and math-related sentences activated more the hand, likely due to the influence of finger counting practice (see also an fRMI follow up by Ghio et al., 2016).

Differently from abstract concepts, for emotional concepts we think that our results render the first explanation more plausible, even if they do not allow to fully disentangle between the two accounts. If we look at the percentages of relations produced (Table 4), we can see that, while abstract concepts elicit a higher percentage of free associations, emotional concepts yield mostly relations involving emotional and interactive aspects.

One further issue remains to be clarified. The influence of pacifier could be occur in different processing phases. The long-term effect we found could be due to the fact that children could not benefit of the linguistic and social input during first encoding in memory of word meanings. Alternatively, it is possible that the effect of pacifier not only influences encoding but is more extended, influencing also consolidation and retrieval. Even if 7-year-olds do not use pacifier, their re-enactment of the word acquisition can be negatively influenced by their previous encoding experience. Further studies are needed to determine in which phase the influence of pacifier occurs.

To summarize, we have demonstrated that in 7-year-olds Concrete, Abstract and Emotional concepts elicit a clearly distinct pattern of conceptual relations. Furthermore, we have shown that the acquisition of the conceptual relations associated to both Abstract and abstract Emotional concepts has an embodied counterpart: it is influenced and modulated by the use of pacifier. Importantly, the influence of pacifier is a long term one. Our results suggest that the influence of pacifier might be due to different mechanisms for the two kinds of concepts—a mechanism ascribing a major role to the linguistic simulation in the case of Abstract concepts, another ascribing a more relevant role to emotional and interactive aspects in the case of abstract Emotional concepts. However, our data do not allow us to conclusively determine which of the two mechanisms is at play; furthermore, the two mechanisms are not necessarily mutually exclusive. Further developmental studies will be needed to investigate more in depth the similarities and differences in the acquisition and representation of different kinds of concepts.

## Ethics statement

This study was carried out in accordance with the recommendations of the Ethic Guidelines of the Institute of Cognitive Sciences and Technologies of the Italian National Research Council, with written informed consent from all subjects. All subjects gave written informed consent in accordance with the Declaration of Helsinki. The protocol was approved by the Ethic Committee of the Institute of Cognitive Sciences and Technologies of the Italian National Research Council.

## Author contributions

LB and AB contributes to the conception and design of the work; the analysis and interpretation of the data and, with CM, to data acquisition. LB and AB contributes to the writing of the manuscript, and CM to revising it.

### Conflict of interest statement

The authors declare that the research was conducted in the absence of any commercial or financial relationships that could be construed as a potential conflict of interest.

## Footnotes

^1^Due to the high presence of zeros in the distribution, the LMM model may not be appropriate to analyze the data, thus we used the Akaike information criterion for model selection. The Akaike information criterion (AIC) is a measure of the relative quality of statistical models for a given set of data. Given a collection of models for the data, AIC estimates the quality of each model, relative to each of the other models. Hence, AIC provides a means for model selection. Among the candidate models, we wanted to select the model that minimized the information loss. The smaller the AIC numeric value, the better the fit. AIC has been calculated using the AIC function in R.

^2^We have run a GLMM model including psycholinguistic variables as covariates, that is the fixed-factors of interest and their interaction (Concept Type ^*^Pacifier) and 7 covariates (i.e., Written Frequency, Imageability, Familiarity, Context Availability, Age of Acquisition, Mode of Acquisition, word Length in letters). First, the psycholinguistic variables have been rescaled using the “scale” function. Results are in line with the GLMM model without covariates (significant Abstract—Concrete concepts difference (β_Concrete_ = 2.51, z-value = 2.6, *p* < 0.001), and Abstract—Emotional concepts difference (β_Emotional_ = 1.36, z-value = 2.05, p < 0.05). No differences between Concrete - Emotional concepts (β_Concrete:Emotional_ = − 0.82, z-value = −1.23, ns), nor between different frequencies of pacifier use (z-values < 1). Neither the Concept type per Pacifier interaction was significant, except for the contrast Concrete vs. Pacifier Two (β_Concrete:PacifierTwo_ = −1.3, z-value = −2.12, *p* < 0.05). Finally, we have performed models comparison using the ANOVA function, which revealed that adding the covariates is not that informative as the two models are not statistically different (Chi-squared = 6.69, n.s.)
