# Supplementary material for: Pacifier Overuse and Conceptual Relations of Abstract and Emotional Concepts
Source: Front Psychol. 2017 Dec 1;8:2014. doi: 10.3389/fpsyg.2017.02014 (PMC5717369; doi:10.3389/fpsyg.2017.02014)
Supplement: Supplementary file 1 [file Table1.doc]

**Preliminary study: Emotionality ratings**

The aim of this preliminary study was to collect data on emotionality values of a list of stimuli among which to successively select experimental materials for subsequent experiments. Due to the absence of standardized data on Emotionality an on-line questionnaire has been used to obtain ratings of emotional content for a list of Italian words.

**1. Method**

**1.1 Participants**

Ratings of Emotionality were obtained from a sample of 30 participants (17 male, mean age 30 years), native speakers of Italian. Participants were contacted via email and, following Informed Consent, they were provided with the link for the completion of the questionnaire. The study was approved by the Research Ethics Committee of the Institute of Cognitive Sciences and Technologies of the Italian National Research Council.

**1.2 Materials and procedure**

We created a list of 252 words by merging the lexical items shared by Della Rosa et al., (2011) and Marconi et al. (1993) databases. The Della Rosa et al. (2011) database provides measures of Concreteness, Imageability, Context Availability, Familiarity, Age of Acquisition and Mode of Acquisition. The Marconi et al. (1993) database provides measures of ‘child written frequency’; the frequency measures are gathered from books read by elementary school children.

Participants of this study were asked to rate the list of words based on a 7-point Likert scale, judging 'to what extent they are able to evoke feelings and emotions. Some words can be highly emotional (e.g., 'anger' and 'joy'), others are poorly emotional (e.g., 'sentence', 'pen'), others have only a few emotional connotations (e.g., 'aunt' and 'singing').' They were asked to assign 1 to poorly emotional words, and 7 to highly emotional words (see also Borghi & Zarcone (2016) for similar survey procedure). The order of the words within the list was automatically randomized for each participants.

Questionnaires were created with Google Forms, a free tool to create and analyze surveys.

**2 Results**

Mean Emotionality values were computed for each items, then multiplied by 100 to produce a range from 100 to 700 (see Della Rosa et al., 2010). Table 1 lists the variables included in the study, and descriptive statistics for each variable are also presented.

Insert **Table 1** here

Pearson correlation among variables was computed and visualized in R with ‘corrplot’ package. Figure 1 presents a graphical display of the correlation matrix.

Insert **Figure 1** here.

Focusing on the correlation of the ratings of emotional content with other variables, Emotionality was positively correlated with Abstractness (r = .50, p-value < .001), Mode of Acquisition (r = .30, p-value < .001) and Age of Acquisition (r = .29, p-value < .001). That is, higher values of Emotionality corresponded to higher values of abstractness and mode of acquisition (i.e., words mainly acquired through language), and highly emotional words were typically acquired later in life. Emotionality was negatively correlated with Concreteness (r = -.50, p-value < .001) and Imageability (r = -.47, p-value < .001), so that highly emotional words were less concrete and imaginable. Finally, Emotionality was not correlated with word’s written frequency and length in letters.

The most important results show that Concreteness is positively correlated with Imageability and Contextual Availability (CA), is slightly correlated with Emotionality, while it is negatively correlated with Age of Acquisition (concepts acquired later are less concrete), Abstractness and Modality of Acquisition (perceptually acquired concepts are more concrete). Frequency, Familiarity and Length are only slightly correlated with the other dimensions.

A subset of this list has been extracted to be used in the experiment.

**Table 1**. Descriptive statistics of the variables

|  | **Mean** | **SD** | **Min** | **Max** |
| --- | --- | --- | --- | --- |
| **IMA** | 525.36 | 166.81 | 166.81 | 700.00 |
| **CONC** | 499.33 | 185.76 | 185.76 | 700.00 |
| **FAM** | 564.13 | 83.04 | 83.04 | 700.00 |
| **AoA** | 282.07 | 93.83 | 93.83 | 525.00 |
| **CA** | 553.06 | 109.30 | 109.30 | 700.00 |
| **ABS** | 281.42 | 171.05 | 171.05 | 635.00 |
| **MoA** | 340.41 | 144.55 | 144.55 | 663.16 |
| **Length** | 721.83 | 205.60 | 300.00 | 1400.00 |
| **EMO** | 333.90 | 141.23 | 0.00 | 636.67 |

*Note*. IMA: imageability (ratings on a 7 point scale, multiplied by 100); CONC: concreteness (ratings on a 7 point scale); FAM: familiarity (ratings on a 7 point scale); AoA: word age of acquisition(ratings on a 7 point scale); CA: Context Availability (ratings on a 7 point scale); ABS: abstractness (ratings on a 7 point scale); Length in letters; MoA: concept mode of acquisition (ratings on a 7 point scale); Length: word length in letters. EMO: words emotionality (ratings on a 7 point scale).

**Figure captions and legends**

**Figure 1.** Plot of correlation matrix.

*Note*. Positive correlations are displayed in blue and negative correlations in red color. Color intensity and the size of the circle are proportional to the correlation coefficients. In the right side of the plot, the legend colors shows the correlation coefficients and the corresponding colors. Correlations with p-values > .01 are leaved blank.
