# Supplementary figures and images for: Pacifier Overuse and Conceptual Relations of Abstract and Emotional Concepts
Source: Front Psychol. 2017 Dec 1;8:2014. doi: 10.3389/fpsyg.2017.02014 (PMC5717369; doi:10.3389/fpsyg.2017.02014)

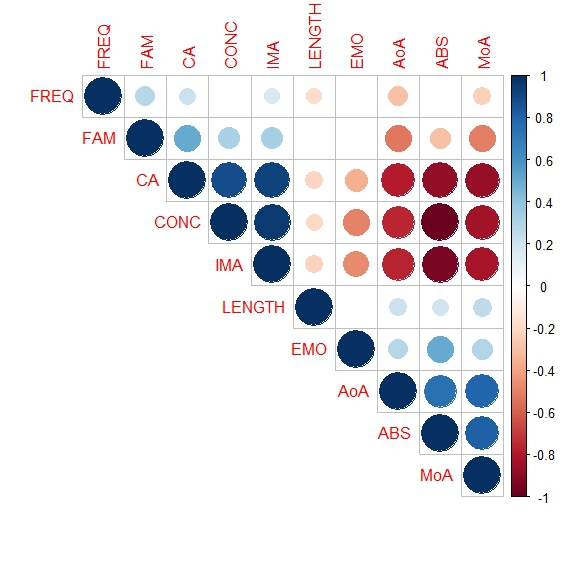

Supplement: Supplementary file 2 [file Image1.JPEG]
