# Supplementary material for: Pacifier Overuse and Conceptual Relations of Abstract and Emotional Concepts
Source: Front Psychol. 2017 Dec 1;8:2014. doi: 10.3389/fpsyg.2017.02014 (PMC5717369; doi:10.3389/fpsyg.2017.02014)
Supplement: Supplementary file 3 [file Appendix1.doc]

**Appendix 1**. Experimental stimuli

| **Concepts type** | **Word (English translation)** |
| --- | --- |
| abstract | accordo (agreement) |
| abstract | bellezza (beauty) |
| abstract | crescita (growth) |
| abstract | cultura (culture) |
| abstract | fantasma (ghost) |
| abstract | inizio (beginning) |
| abstract | mistero (mistery) |
| abstract | numero (number) |
| abstract | scoperta (discovery) |
| abstract | vittoria (victory) |
| concrete | bandiera (flag) |
| concrete | campeggio (camping site) |
| concrete | casco (helmet) |
| concrete | corona (crown) |
| concrete | elicottero (helicopter) |
| concrete | libreria (library) |
| concrete | noce (walnut) |
| concrete | orologio (clock) |
| concrete | pennello (brush) |
| concrete | scatola (box) |
| emotional | amore (love) |
| emotional | bacio (kiss) |
| emotional | cuore (heart) |
| emotional | dolore (pain) |
| emotional | felicita` (happiness) |
| emotional | odio (hate) |
| emotional | paura (fear) |
| emotional | simpatia (sympathy) |
| emotional | sorpresa (surprise) |
| emotional | vergogna (shame) |
