# Supplementary material for: Pacifier Overuse and Conceptual Relations of Abstract and Emotional Concepts
Source: Front Psychol. 2017 Dec 1;8:2014. doi: 10.3389/fpsyg.2017.02014 (PMC5717369; doi:10.3389/fpsyg.2017.02014)
Supplement: Supplementary file 4 [file Appendix2.doc]

**Appendix 2**. Demographic characteristics of the sample of participants

(those marked with an asterisk were not included in the Definition task).

| **Participant** | **Pacifier** | **Gender** | **Age (months)** | **Schooling - mother** | **Schooling -phater** | **Exposure to other languages** |
| --- | --- | --- | --- | --- | --- | --- |
| **1** | Never | Male | 73 | High School | High School |  |
| **2** | Never | Female | 80 | Middle School | Middle School | US English |
| **3** | TwoThree | Male | 80 | High School | High School |  |
| **4** | TwoThree | Female | 74 | High School | Middle School |  |
| **5** | TwoThree | Female | 82 | High School | Middle School |  |
| **6** | TwoThree | Female | 69 | High School | High School |  |
| **7** | TwoThree | Male | 75 | University | University |  |
| **8** | Never | Female | 74 | High School | High School | Cingalese |
| **9*** | NA | Female | 76 | High School | High School |  |
| **10** | TwoThree | Male | 72 | NA | NA |  |
| **11** | Three | Male | 80 | Middle School | University |  |
| **12** | Two | Female | 74 | Middle School | Middle School |  |
| **13** | Three | Female | 80 | Middle School | Middle School |  |
| **14*** | NA | Female | 94 | High School | High School |  |
| **15** | Two | Female | 83 | University | University |  |
| **16** | Two | Female | 69 | High School | High School |  |
| **17** | TwoThree | Male | 83 | Middle School | Middle School |  |
| **18** | TwoThree | Male | 75 | Middle School | High School | Portuguese |
| **19*** | NA | Male | 76 | High School | High School | Spanish |
| **20** | Two | Male | 76 | High School | High School |  |
| **21** | Two | Male | 74 | University | High School |  |
| **22*** | NA | Female | 73 | University | High School |  |
| **23*** | NA | Female | 82 | University | High School |  |
| **24** | Two | Male | 74 | University | University | English |
| **25** | TwoThree | Male | 82 | University | High School | English |
| **26** | Two | Male | 76 | University | High School | Moldovan |
| **27** | Two | Male | 83 | University | High School |  |
| **28** | Three | Male | 72 | High School | High School | Portuguese |
| **29** | TwoThree | Female | 79 | Middle School | High School |  |
| **30** | Two | Male | 81 | University | University |  |
| **31** | TwoThree | Female | 83 | High School | Elementary School |  |
| **32** | TwoThree | Female | 75 | Middle School | Middle School |  |
| **33** | Two | Male | 76 | University | NA |  |
| **34** | Never | Male | 77 | High School | Elementary School | Pakistani |
| **35*** | NA | Female | 76 | High School | High School | Arabic |
| **36** | TwoThree | Female | 81 | High School | High School |  |
| **37** | Three | Male | 77 | University | University |  |
| **38** | TwoThree | Male | 83 | High School | High School |  |
| **39** | Two | Male | 76 | High School | High School |  |
| **40** | TwoThree | Male | 75 | High School | High School | German |
| **41** | TwoThree | Female | 75 | University | High School | Spanish |
| **42*** | NA | Female | 83 | High School | High School |  |
| **43** | Three | Female | 77 | University | University |  |
| **44** | Never | Male | 83 | University | High School |  |
| **45** | TwoThree | Male | 76 | High School | High School |  |
| **46*** | NA | Male | 74 | University | University | Arabic |
| **47** | Two | Female | 76 | High School | Middle School |  |
| **48*** | NA | Female | 77 | University | High School |  |
| **49** | Two | Female | 72 | NA | NA | Albanian |
| **50*** | NA | Male | 72 | High School | Middle School |  |
| **51** | TwoThree | Female | 76 | High School | High School |  |
| **52** | Two | Female | 83 | Middle School | High School |  |
| **53** | Two | Female | 79 | High School | High School |  |
| **54*** | NA | Female | 69 | High School | High School |  |
| **55*** | NA | Female | 72 | NA | NA | Romanian |
| **56** | Two | Male | 82 | High School | High School | Chinese |
| **57** | Two | Male | 76 | High School | High School |  |
| **58** | Never | Female | 83 | University | University |  |
| **59*** | NA | Female | 83 | University | High School |  |
